# “What Is eHealth”: Time for An Update?

**DOI:** 10.2196/resprot.4065

**Published:** 2015-03-12

**Authors:** Emiel A Boogerd, Tessa Arts, Lucien JLPG Engelen, Tom H van de Belt

**Affiliations:** ^1^Radboud University Medical CenterDepartment of Medical PsychologyNijmegenNetherlands; ^2^Radboud University Medical CenterRadboud REshape Innovation CenterNijmegenNetherlands

## Abstract

The annual number of articles reporting on eHealth interventions has increased over the last 10 years. In contrast, the last article in this journal on the definition of eHealth was published in 2006. This leads to the question whether the field itself has reached consensus about the definition and description of eHealth or whether it is in need for a new review of the literature and a new description of the rapidly changing field of eHealth. Since the JMIR community has successfully collaborated on the “CONSORT-eHealth” in the past, we would like to use the same strategy to explore the need for a new definition of eHealth and the creation of a taxonomy for this field. Therefore, we hereby submit a call to all JMIR-readers, to fill out a 4-question survey on their ideas about a refined eHealth definition. Based on these results, we will decide whether or not to engage in a systematic review. Logically, the entire JMIR community is invited to join us in our attempt to further elucidate the field of eHealth.

In 2001, the editor of the Journal of Medical Internet Research, Gunther Eysenbach, reported on the need for defining eHealth [1]. In what would be the first article of the “What is eHealth” series, he defined eHealth as follows:

eHealth is an emerging field in the intersection of medical informatics, public health and business, referring to health services and information delivered or enhanced through the Internet and related technologies. In a broader sense, the term characterizes not only a technical development, but also a state-of-mind, a way of thinking, an attitude, and a commitment for networked, global thinking, to improve health care locally, regionally, and worldwide by using information and communication technology.

Subsequently, Eysenbach invited researchers to explicate their views on the definition of, which together would elucidate the realm of eHealth. This invitation led to a series of papers reporting about definitions concerning eHealth.

In reaction to Eysenbach’s invitation, Della Mea [2] described eHealth as a popular term which scientists have adopted from the fields of commerce and economics. Instead of providing a uniform definition, Della Mea described eHealth as a broad term that encompasses multiple domains. In the following years, the field of eHealth expanded and the number of eHealth related studies increased (Figure 1). 

**Figure 1:**
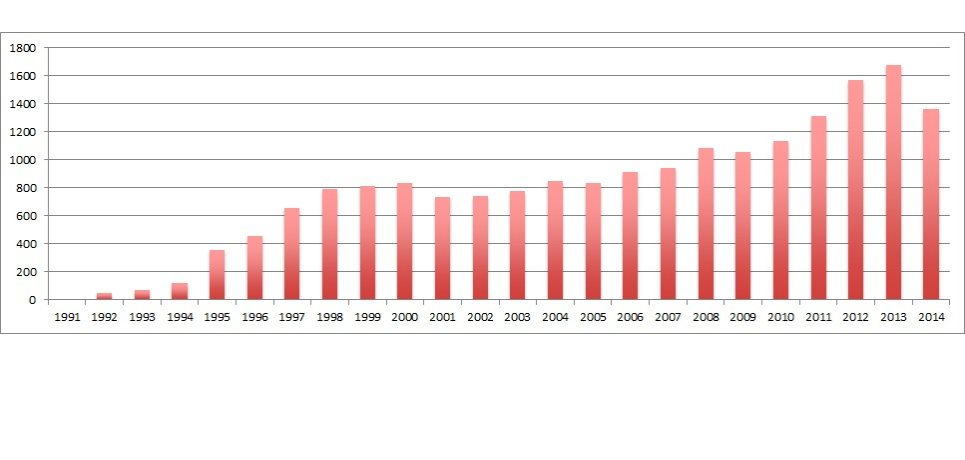
Number of papers mentioning eHealth per year in Pubmed (search conducted in fall 2014, 2014 is therefore incomplete).

However, except for Eysenbach’s broad definition of 2001, a clear uniform and comprehensive definition of eHealth and its domains was still lacking. Four years had passed when, in 2005, Oh et al [3] pointed at the problem which arose from the lack of a definition for eHealth: How can we communicate about a phenomenon when that phenomenon is not clearly defined? In a qualitative, systematic review, they found 51 unique definitions for eHealth. Although health and technology were mentioned in all 51 definitions, a uniform description of these general terms were missing. In 2005, Pagliari et al [4] referred to the same problem (the lack of a clear and uniform definition) in the light of archiving and retrieving eHealth studies. In their qualitative study, Pagliari and colleagues found 36 different definitions. They discovered that most definitions applied to the functional scope of eHealth rather than to specific applications. Based on their findings, the authors concluded that the definition posted by Eysenbach sufficed, although they made some adjustments:

eHealth is an emerging field of medical informatics, referring to the organization and delivery of health services and information using the Internet and related technologies. In a broader sense, the term characterizes not only a technical development, but also a new way of working, an attitude, and a commitment for networked, global thinking, to improve health care locally, regionally, and worldwide by using information and communication technology.

In contrast to their fellow “What is eHealth?” contributors, who concentrated on the definition of eHealth, Jones et al [5] published an article in 2005, in which they described stakeholders’ views on the concerns and promise of eHealth in future research. They discovered that the views of the various stakeholders were, surprisingly, not that different; their main recommendations were that the scope of eHealth research should be on using, processing, sharing, and controlling information. Unfortunately, it did not lead to a new definition, which was also noticed by Ahern et al [6]. In this most recent article in the “What is eHealth” series, which was published in 2006, the authors clearly underscore the need for a more coordinated and rigorous attempt to define the field of eHealth [6].

In addition to the wish for a uniform definition of eHealth, the availability of related terms such as Medicine 2.0, Web 2.0, Health 2.0, mHealth, Telecare and Telehealth may be confusing. Although not identical, there seems to be a lot of overlap, and different terms are used interchangeably throughout literature [6]. The articles in the “What is eHealth” series, point out that eHealth related research encompasses a broad field that ranges from theory development to large randomized controlled trials. What’s more, usage of eHealth differs per health care setting or even per person, varying from interventions or services such as apps, websites, online discussion groups to real-life medical data collection, for instance by using wearables. This supports the need for a clear uniform description of eHealth and an attempt to compose a comprehensive overview of the various domains in the eHealth field.  As such, it would be very interesting to investigate whether a taxonomy of the broad field of eHealth, including related topics, would lead to a better definition of the various domains within eHealth.

The annual number of articles reporting on eHealth interventions has increased over the last 10 years (see Figure 1). In contrast, the last article in this journal on the definition of eHealth was published in 2006. This leads to the question whether the field itself has reached consensus about the definition and description of eHealth or whether it is in need for a new review of the literature and a new description of the rapidly changing field of eHealth. Since the JMIR community has successfully collaborated on the “CONSORT-eHealth” in the past, we would like to use the same strategy to explore the need for a new definition of eHealth and the creation of a taxonomy for this field. Therefore, we hereby submit a call to all JMIR-readers, to fill out a 4-question survey on their ideas about a refined eHealth definition. The results of this survey will be published in the Journal of Medical Internet Research. Based on these results, we will decide whether or not to engage in a systematic review. Logically, the entire JMIR community is invited to join us in our attempt to further elucidate the field of eHealth.

## Survey

http://tinyurl.com/eHealthdef
